# From Discomfort to Distress: A Critical Analysis of Hyperemesis Gravidarum in the Emergency Room

**DOI:** 10.7759/cureus.44004

**Published:** 2023-08-23

**Authors:** Arushi Joshi, Garima Chadha, Palaniappan Narayanan

**Affiliations:** 1 Medicine, Sri Ramachandra Institute of Higher Education and Research, Chennai, IND; 2 Emergency Medicine, Sri Ramachandra Institute of Higher Education and Research, Chennai, IND; 3 Obstetrics and Gynecology, Sri Ramachandra Institute of Higher Education and Research, Chennai, IND

**Keywords:** first trimester complications, nausea and vomiting, pregnancy, emergency room, hyperemesis gravidarum

## Abstract

Hyperemesis gravidarum (HG) is a severe and debilitating condition characterized by persistent and excessive nausea and vomiting during pregnancy (NVP), often leading to significant maternal and fetal morbidity. This literature review aims to provide a scientifically comprehensive overview of HG within the context of the emergency room (ER) setting. This review aims to enhance understanding and improve the management of HG cases presented to the ER by synthesizing current knowledge and evidence-based practices.

This literature review encompasses a systematic analysis of relevant scientific literature, encompassing original research studies, review articles, and clinical guidelines. An extensive search of electronic databases was conducted, covering the period from January 2003 to January 2023. Keywords related to HG, pregnancy-related complications, emergency medicine, and ER management were employed to identify pertinent publications.

Through the literature review, we found the incidence of HG-related ER admission to be 0.8%. Although the etiology of HG remains to be unknown, a strong association was found between developing HG in pregnant females and a history of gastrointestinal (GI) disorders, use of cannabis, and pregnancies conceived through artificial reproductive technology (ART). Furthermore, overweight females were more likely to develop HG. Maternal smoking was found to be protective against HG. The symptoms of HG mainly include intractable nausea and vomiting occurring usually between four and nine weeks of gestational age with a significant aversion to food and loss of weight. Diagnosis is done through a strong clinical suspicion, a history of HG in previous pregnancies, and a basic metabolic panel. Treatment includes intravenous (IV) fluids, antiemetic therapy, corticoids, thiamine supplements, and laxatives. In our review, we highlight a few complications that can be seen in HG through a synopsis of unique case reports found during our literature search.

In conclusion, through this review, we wish to highlight HG as an obstetrical emergency. We aim to improve understanding, enhance early recognition, and promote evidence-based management strategies for HG in the emergency room. We hope that the findings presented herein will serve as a valuable resource for healthcare professionals, researchers, and policymakers involved in the care of pregnant females experiencing HG in the ER.

## Introduction and background

Hyperemesis gravidarum (HG) is a condition in which there is intractable vomiting and nausea in pregnant females, which may require urgent hospitalization. This condition occurs mainly in the first trimester of pregnancy, with only about 10% of cases extending beyond 20 weeks [1], and results in a loss of greater than 5% of the total body weight. The incidence of the condition is 0.8%-2.3% [2,3], with many pregnant females presenting in the emergency department (ED) due to dehydration and malnutrition. According to some studies, the admission rate for a patient presenting with hyperemesis gravidarum is 0.8% [4].

## Review

Etiology

The etiology of HG is largely unknown, but recent studies indicate a strong genetic component; it is suggested that the risk of HG in offspring increases by nearly threefold if the maternal history of HG is present [5]. Other genomic studies indicate an association of growth and differentiation factor 15 protein (GDF15), a known cause of cancer cachexia, with HG [6].

While other causes of HG are not as well researched, there is evidence to show that females who have undergone artificial reproductive technology (ART) for pregnancy have a higher incidence of HG [4]. Other suggested causes include a history of motion sickness or gastrointestinal (GI) disorders, *Helicobacter pylori* infections, and low maternal weight before pregnancy [7].

Extremes in body weight in either direction are associated with an increased incidence of HG. All factors being similar, overweight females are more likely to develop hyperemesis gravidarum than their underweight counterparts.

Maternal prepregnant smoking was found to be protective against HG. Among females who smoke daily or occasionally, the prevalence of HG is lower than in nonsmokers. The smoking habits of the partner are not found to have any effect [8]. Cannabis, often used as an antiemetic, can also contribute to nausea and vomiting during pregnancy (NVP) due to its biphasic effect: If taken in acute, low doses, it has an antiemetic effect, while high doses or chronic use can cause cannabinoid hyperemesis syndrome, leading to increased intractable vomiting [8].

This can be especially dangerous, as some females may take cannabis during pregnancy to reduce nausea in the first trimester. A study from North Carolina shows almost 20% use of cannabis in females with self-reported mild to severe nausea while only 4.5% use in asymptomatic females [9].

Clinical features

Nausea and vomiting during pregnancy (NVP) occurs in 50%-90% of pregnancies, with nausea and vomiting in approximately 50%-55% and nausea alone in 25%. Although NVP has been commonly referred to as "morning sickness," nausea can occur at any time of the day, last for varying periods, and occur with or without episodes of vomiting. The usual onset for NVP is between four and nine weeks of gestational age, with maximal symptoms at 12-15 weeks and resolution by 20 weeks of gestational age. In a study conducted to assess the emergency department burden of hyperemesis gravidarum in the United States from 2006 to 2014, HG was the third most common reason, after threatened abortion and genitourinary tract infection, for early pregnancy ED visits, with 7.2% of those visits having reported HG as the primary diagnosis, and accounted as the fourth most common reason for admission for pregnant patients [10]. The symptoms of HG predominantly include any combination of the following: nausea, gagging, retching, dry heaving, vomiting, and odor and/or food aversion. Each female usually has a certain precipitating factor that triggers nausea and vomiting, that is, movement-induced, heartburn, food, and/or odor triggers [11].

Diagnosis in the emergency room (ER)/admission

NVP can have several causes, and HG is mainly a clinical diagnosis of exclusion. The mainstay of the clinical workup for patients with hyperemesis gravidarum is a detailed evaluation to exclude other potential causes of presenting symptoms. The following laboratory tests are most included in the initial assessment: complete blood count and basic metabolic panel, urinalysis for ketones and specific gravity, thyroid function studies, amylase/lipase levels, and, in early pregnancies, a serum beta-human chorionic gonadotropin (hCG) level for the evaluation of possible molar or multiple gestations [12]. However, these markers have been reported to be unreliable indicators for the diagnosis and severity of HG [13]. In a study done to determine the association of inflammatory markers with the presence and severity of HG, it was found that neutrophil-to-lymphocyte ratio (NLR) and high-sensitivity C-reactive protein (hsCRP) levels are increased in HG disease compared to gestational age-matched control group subjects. Furthermore, NLR and hsCRP values were correlated with the severity of disease. Thus, NLR could be used as a marker for both the presence and severity of hyperemesis gravidarum [14]. Thus, affordable and acceptable markers such as NLR and platelet-to-lymphocyte ratio (PLR) could be used in the evaluation of HG patients in emergency settings [15].

The differential diagnosis to consider for nausea and vomiting during pregnancy includes peptic ulcers, gastroenteritis, cholecystitis, hepatitis, genitourinary conditions such as urinary tract infections (UTI), drug-induced vomiting, and neurological conditions. A well-focused history and examination can help eliminate most differentials in a patient [16].

Management in the ER

The literature on the management of HG in the ER is sparse, and most patients that present to the ER with suspected HG are given supportive treatment focusing on fluid, vitamin, and electrolyte replacement, as well as antiemetic therapy [17].

The American College of Obstetricians and Gynecologists (ACOG) in the practice bulletin number 189 recommends the use of the Pregnancy-Unique Quantification of Emesis and Nausea (PUQE) questionnaire to assesses the severity of nausea and vomiting during pregnancy in the first trimester as shown in Table 1.

**Table 1:** PUQE score PUQE: Pregnancy-Unique Quantification of Emesis and Nausea

	1 point	2 points	3 points	4 points	5 points
Duration of nausea in the past 12 hours	0	<1 hour	2-3 hours	4-6 hours	>6 hours
Number of vomiting episodes in the past 12 hours	0	1-2 hours	3-4 hours	5-6 hours	≥7 hours
Number of episodes of dry heaves in the past 12 hours	0	1-2 hours	3-4 hours	5-6 hours	≥7 hours

**Figure 1:**
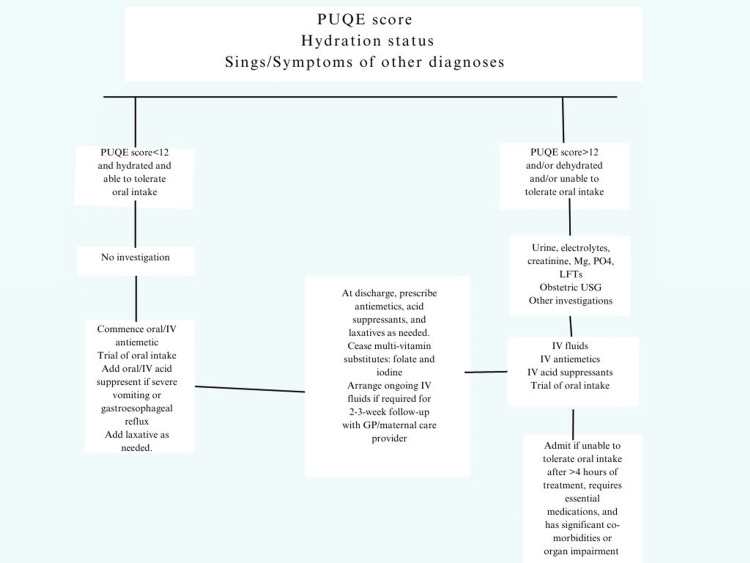
Treatment algorithm for HG PUQE, Pregnancy-Unique Quantification of Emesis and Nausea; IV, intravenous; prn, as needed; GP, general practitioner; LFTs, liver function tests; USG, ultrasonography; HG, hyperemesis gravidarum Lowe SA, Steinweg KE: Review article: management of hyperemesis gravidarum and nausea and vomiting in pregnancy. Emerg Med Australas. 2022, 34:9-15. 10.1111/1742-6723.13909 [18]

The female's PUQE score, level of hydration, and clinical condition can be used in combination by the clinician to assess the patient and formulate a treatment plan; the treatment algorithm is as shown in Figure 1.

Intravenous (IV) fluids

The initial treatment of HG in ER consists of giving the patient intravenous (IV) fluids, which have the dual benefit of replacements of fluid volume and an anti-nausea effect [19]. Normal saline (NS) is generally the preferred fluid for replacement therapy. Additional electrolytes can be given if electrolyte abnormalities are noted in the blood investigations. Severe hyponatremia should be corrected no faster than 10 mmol/L/day as rapid correction can lead to central pontine myelinolysis [20]. Dextrose is generally not indicated in pregnant females as it has shown no additional benefits over normal saline (NS) [21]. Moreover, the use of dextrose must be judicious in pregnant patients, as it may precipitate Wernicke's encephalopathy in thiamine-deficient patients [22].

Antiemetic therapy

Antiemetic therapy is the mainstay of treatment of HG, but their use in pregnant females is sometimes debated or restricted due to the perceived increased risk of teratogenicity. Despite this, multiple drugs have been deemed by teratologists to be safe for use in the first trimester in the treatment of HG including, but not limited to, antihistamines such as promethazine, ondansetron, and metoclopramide [23]. It should also be noted that multiple studies have found that the outcomes of pregnancy were better in females with HG who took antiemetic therapy as opposed to those with HG who did not [24]. The Oxford Handbook of Emergency Medicine (Fifth Edition) suggests the use of IV or intramuscular (IM) cyclizine (50 mg twice or thrice daily) or prochlorperazine (25 mg thrice or four times daily, up to maximum of 100 mg/day) as the treatment of choice for hyperemesis in pregnancy. Metoclopramide is also considered a good first-line drug for HG and can be given at 0.5 mg/kg (maximum of 30 mg/day). Some studies indicate metoclopramide as the drug of choice [24]over antihistamines or phenothiazines. If HG is not controlled by the above drugs, ondansetron can be given as a second-line treatment. The use of ondansetron should be avoided in females with preexisting QT prolongation. Antiemetic drugs should not be used concurrently in females with HG. If one drug fails to provide control over vomiting, it should be ceased, and an alternate first-line drug or second-line drug should be started.

Corticosteroids

Corticosteroids are considered third-line therapy for HG due to some inconsistent studies that have shown a link between their use in the first trimester and the development of orofacial abnormalities of the fetus [25,26],although this has not been confirmed by other subsequent studies. Due to the obvious benefit provided, many teratologists still support their use in pregnancy [27,28]. Hydrocortisone or prednisolone are the drugs of choice as they are inactivated in the placenta. Dexamethasone should be avoided as it can cross the placenta and lead to fetal adrenal suppression [29].

Thiamine supplementation

Excess vomiting can also lead to thiamine deficiency, which may further lead to Wernicke's encephalopathy; pregnant females are at an increased risk as they already have an increased thiamine requirement during their gestational period. Initial treatment in HG is done by intravenous supplementation, with later oral dosing prescribed for outpatient follow-up as required^ ^[30].

Laxatives

Poor oral intake due to nausea, hypomotility of the GI tract due to pregnancy, and the use of certain drugs such as ondansetron may cause constipation in females. As such, preemptive action can be taken in stable HG patients with the use of laxatives to prevent constipation. While the complete resolution of symptoms may not occur in the ER, if the female has tolerated the pharmacotherapy well and is able to demonstrate the ability to eat and drink, vomiting has subsided, and all concurrent conditions have been managed, she may be discharged from the ER. Conversely, in severe cases where the patient is hemodynamically unstable and there is severe electrolyte imbalance, renal impairment or acute kidney injury (AKI) (creatinine of >90 mmol/L), or a presence of starvation ketoacidosis, the admission of the patients under the obstetrics and gynecology (OB/GYN) department is warranted. Ketonuria is no longer considered to have any association with the severity, course, or outcome of HG [31], and decisions on whether to admit a patient should not be based on the levels of ketonuria. The referral process from the ER to the OB can consequently lead to several treatment delays and can significantly contribute to extended inpatient hospitalization. Treatment options for HG are based on individual clinician's preference and usually involve enteral, parenteral treatment or the medical termination of pregnancy [19].

Unique complications of HG presented in the ER

During our literature review, we came across several unique complications occurring due to HG that highlight the timely and intensive management that is required for this pathology. Below, we present a short summarization of a few noteworthy cases.

Subluxation of the Left Eye in a Patient With Hyperemesis Gravidarum

A 36-year-old female G1P0 at 14 weeks of gestation was diagnosed with hyperemesis gravidarum and complained of popping sensation in the left eye. On examination, proptosis of the left eye was present, and under analgesia, left eye reduction was done with the return of full extraocular movements, and the patient was discharged with conservative management but returned due to another episode of the same complaints. The patient was then admitted overnight, and left lateral tarsorrhaphy was done the next morning [32].

Spontaneous Perforation of Choledochal Cyst and Hyperemesis Gravidarum

A 28-year-old primi complaining of vomiting for five days presented to the emergency room and was admitted. On day 2 of admission to the hospital, abdominal distention was present, and an ultrasound showed common bile duct (CBD) dilatation. The ascetic fluid was bile stained, and an emergency exploratory laparotomy was done, which showed spontaneous perforation. The patient was managed and carried the pregnancy to term^ ^[33].

Spontaneous Pneumothorax Seen in a Patient With HG

A 21-year-old first trimester primigravida female with hyperemesis gravidarum was noted to have incidental subcutaneous emphysema during thyroid ultrasound. Follow-up radiograph demonstrated supraclavicular subcutaneous emphysema, left apical pneumothorax, and pneumomediastinum. The patient was transferred to the intensive care unit (ICU) and evaluated for esophageal rupture. Swallow studies/endoscopy was normal, and the patient was hemodynamically stable; she was treated conservatively with antibiotics and monitored. The patient's condition improved, and she was discharged on hospital day 6. Subcutaneous emphysema secondary to hyperemesis gravidarum is a rare but potentially life-threatening condition in which the source of the mediastinal leak needs to be immediately determined [34].

Rhabdomyolysis in HG

A 20-year-old female G3P2 at 19 weeks of gestational age came to the ER with complaints of nausea and vomiting. She had a history of hyperemesis gravidarum diagnosed in the first two pregnancies. She had reduced ability to tolerate oral intake in the past three days. On admission in the ER, an ECG was obtained, which showed U waves. She also had decreased levels of potassium phosphate and elevated levels of creatinine kinase (CK). The patient was admitted to the medical ICU and was given IV fluids, and electrolyte correction was done. The patient's creatinine kinase values were monitored closely. She was given folate and vitamin B6 supplementation to improve nausea. After aggressive hydration, electrolyte and CK levels normalized, her weakness resolved, and the patient was discharged [35].

Though not commonly encountered, these complications highlight the need for an aggressive and efficient management of HG starting from the ER.

## Conclusions

It is known that HG is a debilitating condition characterized by severe nausea and vomiting during the first trimester of pregnancy and leads to significant physiological and psychological distress for females. The management of HG in the ER requires a multidisciplinary approach involving obstetricians, nurses, dieticians, and mental health specialists. The review explores the challenges faced by healthcare providers in the ER while managing HG. These include diagnostic dilemmas, limited awareness among medical professionals, and resource constraints. Addressing these challenges necessitates the implementation of standardized protocols, continuous medical education, and improved access to specialized care services. In conclusion, the management of HG in the ER requires a comprehensive understanding of the condition, early recognition, and a multidisciplinary approach. By implementing evidence-based guidelines, improving healthcare provider awareness, and providing appropriate psychological support, we can strive to enhance the care provided to females with HG in the emergency room, ultimately improving maternal and fetal outcomes. Further research is warranted to explore novel treatment modalities, long-term effects of HG, and strategies for prevention and early intervention.
